# The transition from child and adolescent to adult mental health services with a focus on diagnosis progression

**DOI:** 10.1192/bjb.2018.39

**Published:** 2018-10

**Authors:** Ann Collins, Antonio Muñoz-Solomando

**Affiliations:** 1 Tonteg Child and Family Clinic, Cwm Taf University Health Board, Pontypridd, Wales

## Abstract

**Aims and method:**

This article examines mental health disorders as individuals transition from adolescence to adulthood. Data were collected from clinical records of patients who had transitioned from child and adolescent mental health services to adult mental health services in a region in South Wales. Demographics and clinical diagnoses under both services were recorded. Patterns between adolescent and adult disorders as well as comorbidities were investigated using Pearson's χ^2^-test and Fisher's exact test.

**Results:**

Of the 98 patients that transitioned from one service to the other, 74 had changes to their diagnoses. There were 164 total changes to diagnoses, with patients no longer meeting diagnostic criteria for 64 disorders and 100 new disorders being diagnosed. Comorbidity increased in adulthood.

**Clinical implications:**

Diagnoses can evolve, particularly during adolescence and early adulthood. Therefore regular reassessment is paramount for successful treatment.

**Declaration of interest:**

None.

The majority of mental health disorders are identified during adolescence and early adulthood.^1^ By the age of 21, more than half of the population have experienced at least one mental health disorder.^2^^,^^3^ Adolescent mental health disorders also place individuals at an increased risk of further difficulties as adults.^4^^,^^5^ However, this effect has been found to be limited when adolescent episodes are brief, emphasising the need for prompt and effective treatment.^6^ Despite many studies having identified adolescence as a risk period for the onset of mental health disorders, the course these disorders take is not yet fully understood. To date, it is unclear why some individuals develop a recurrent pattern of mental illness, whereas mental illness is confined to the adolescent period for others.

## Patterns of diagnostic transition

As people with mental health disorders transition from adolescence to adulthood, their disorders can follow different trajectories. In some cases, they can be predecessors of a different disorder in adulthood, displaying a heterotypic pattern.^4^^,^^5^^,^^7^^,^^8^ In other cases, they may remain diagnostically stable and follow a homotypic pattern.^1^^,^^4^ It is possible for both a heterotypic and homotypic pattern of transition to be displayed along with the development of comorbid disorders, especially as these are more likely to develop during the adolescent and young-adulthood phase of development.^9^^,^^10^

## Study aims

Using a clinical sample, this study aims to examine mental disorders as people transition from adolescence to adulthood in a population in South Wales, UK. Patterns between adolescent and adult mental health disorders as well as comorbidities were investigated.

## Method

Data were collected in July 2013. Clinical records were examined retrospectively for all patients referred to adult mental health services (AMHS) within Cwm Taf University Health Board over a 5-year period.

### Inclusion criteria

Included participants were aged 15–22 at the time of referral and had a documented history of involvement with child and adolescent mental health services (CAMHS) in the 2-year period prior to referral to adult services.

### Exclusion criteria

People were excluded from the study for the following reasons:

• lack of sufficient engagement with CAMHS or AMHS to allow a full assessment;
• AMHS involvement of less than a year;
• lack of documented diagnosis from CAMHS or AMHS;
• CAMHS service provided by another health board.

### Data collection

Electronic AMHS notes were analysed. CAMHS paper documentation were accessed if required. The data collected included:

• date of referral to AMHS;
• age at time of referral;
• geographical area;
• documented diagnosis in discharge paperwork from CAMHS;
• documented diagnosis in AMHS clinical notes or correspondence.

Diagnoses were recorded as per clinical coding, based on the ICD-10 (1992). In a small number of participants (*n* = 17), no formal ICD-10 diagnosis was made by CAMHS clinicians. Instead, formulation was used to describe the participant's presentation at the time. The term emotional difficulties was used to capture these cases.

Some disorders were grouped into categories. Hyperkinetic disorder, conduct disorder and oppositional defiant disorder (ODD) were classified together as externalising disorders. Generalised anxiety disorder, obsessive–compulsive disorder (OCD) and phobic disorders were classified together as anxiety disorders. Different forms of substance misuse were categorised as mental and behavioural disorders due to psychoactive substance use. These disorders were grouped in this way because of their level of comorbidity within the category. No diagnosis of conduct disorder or ODD presented without comorbid hyperkinetic disorder. Phobic disorder and OCD did not present as single anxiety disorders. Those participants who misused substances, misused at least two. Therefore, we could not make conclusions regarding an individual diagnosis transition for those individual disorders. Listing all the individual disorders led to a multitude of combinations within the categories and a pragmatic decision was made to group the disorders.

### Ethical approval

Support for this research was obtained from the Research and Development Department within Cwm Taf University Health Board, which is responsible for both the AMHS and CAMHS clinical records. The need for ethical approval was discussed extensively but deemed unnecessary for the following reasons: we were trust employees and collected data on site, originally as part of an audit; the method of data collection allowed identifiable information to be anonymised at the point of collection; and the data used in this study were limited to demographic and diagnosis details.

### Statistical analysis

Pearson's χ^2^-test and Fisher's exact test were used for the comparison of frequencies between two discrete variables. All of the reported *P*-values are two tailed. Statistical significance was set at 0.05 and analyses were conducted using the SPSS version 21 statistical software for windows. Standardised residuals (S_*i*_) were calculated where a significant result was found.

## Results

**Fig. 1:**
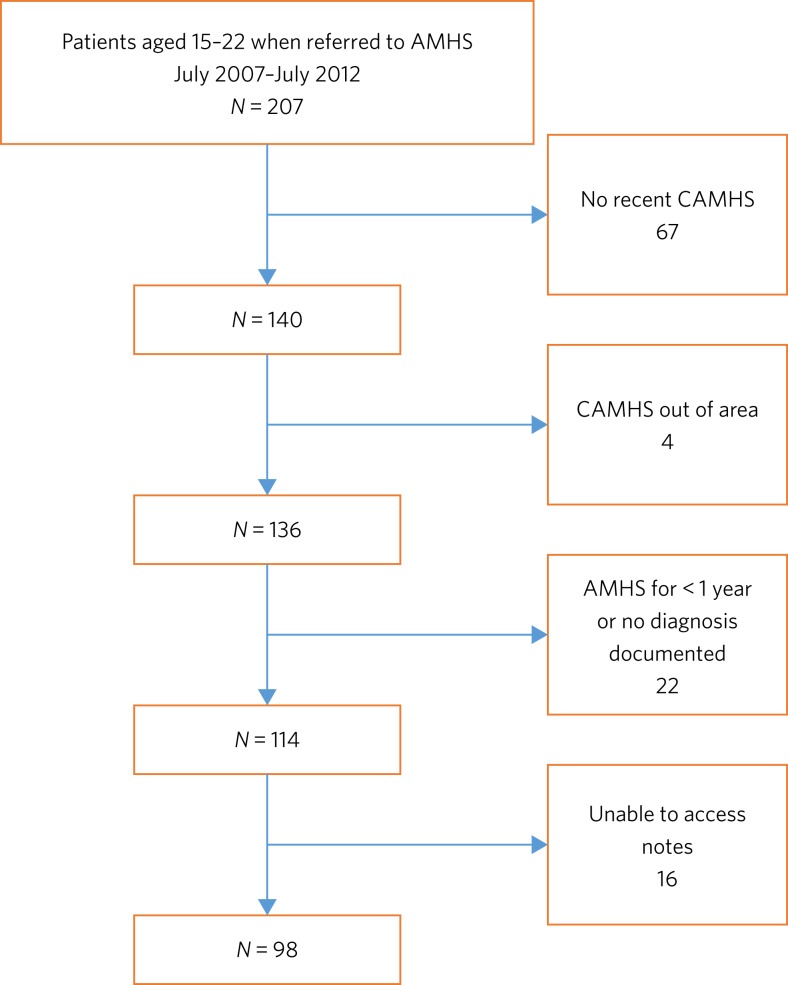
Flow chart illustrating the selection process for participation in the study.

A total of 207 people aged 15–22 were referred to AMHS in the study period. Figure 1 illustrates how the final sample of 98 participants was created. Of the 98 participants, 60 were male (61.2%). The mean age was 18.1 (s.d. 1.46), with an age range of 16–23. There were three sources of referrals to AMHS: 60 participants (61%) were referred by their general practitioner, 37 (38%) by CAMHS and 1 (1%) by the AMHS Crisis Resolution Team. There were 42 participants (43%) who had been referred within 1–12 months of 1 July 2012; 19 (19%) were referred within 13–24 months; and 19 (19%) were referred within 25–36 months, 9 (9%) within 37–48 months and 9 (9%) within 49–60 months.

The CAMHS disorders were categorised into 13 diagnostic categories. A total of 65 participants (66%) had a singular diagnosis, 29 (30%) had two diagnoses and 4 (4%) had three diagnoses. This led to 135 individual diagnoses. The most common diagnostic category in CAMHS was the externalising disorders. The AMHS disorders were also categorised into 13 diagnostic categories, 10 of which were present in the CAMHS sample. A total of 46 participants (47%) had a singular diagnosis, 37 (38%) had two diagnoses, 14 (14%) had three and 1 (1%) had four diagnoses. This led to 166 individual diagnoses. Externalising disorders remained the most prevalent diagnosis. Frequencies of individual diagnostic categories are detailed in Table 1.

**Table 1:** Frequencies of individual diagnostic categories

Clinical code of disorders (ICD-10 codes)	CAMHS frequency (%)	AMHS frequency (%)
Bipolar affective disorder (F31)	2 (1)	5 (3)
Depression (F32)	19 (14)	30 (18)
Elective mutism (F94)	1 (1)	0 (0)
Dissocial personality disorder (F60.2)	0 (0)	8 (5)
Anorexia nervosa (F50)	5 (4)	0 (0)
Emotional difficulties	17 (13)	0 (0)
Emotionally unstable personality disorder (F60.3)	0 (0)	12 (7)
Externalising disorder (F90–91)	57 (42)	38 (23)
Learning disability (F81.9)	1 (1)	1 (1)
Neurotic disorder (F40–42)	14 (10)	23 (14)
Mental and behavioural disorder due to psychoactive substance use (F10–19)	5 (4)	25 (15)
Mental disorder due to brain damage or dysfunction (F06)	0 (0)	1 (1)
Pervasive developmental disorder (F84)	8 (6)	7 (4)
Schizophrenia (F20)	4 (3)	6 (4)
Acute and transient psychotic disorders (F23)	1 (1)	9 (7)
Tic disorder (F95)	1 (1)	1 (1)

When the diagnoses of participants in CAMHS were compared with the diagnoses in AMHS, 164 changes (mean 1.65, s.d. 1.15) were found. Of the disorders diagnosed by CAMHS, 64 were no longer present after participants transferred to AMHS, and 100 new disorders were diagnosed in AMHS. Of the 98 participants, 23 (23%) had no changes to their diagnosis.

Disorders diagnosed in CAMHS were examined retrospectively, observing how these cases developed diagnostically under AMHS. Heterotypic continuity was observed to be significant in three disorder categories. These categories were CAMHS externalising disorders to dissocial personality disorders (DPP) (*P* = 0.01), CAMHS eating disorders to emotionally unstable personality disorders (EUPD) (*P* = 0.01) and CAMHS emotional difficulties to EUPD (*P* = 0.01). Homotypic continuity was evident for externalising disorders (χ^2^ = 37.5, *P* < 0.001), neurotic disorders (χ^2^ = 6.04, *P* = 0.01), bipolar affective disorder (*P* < 0.001), pervasive developmental disorder (*P* < 0.001), mental and behavioural disorder due to psychoactive substance use (*P* = 0.01) and Tourette's syndrome (*P* = 0.01). Several disorders displayed an inverse relationship and, if present in CAMHS, the presence of other disorders in AMHS decreased. This was the case for the following diagnoses: CAMHS neurotic disorder and AMHS externalising disorder (χ^2^ = 5.47, *P* = 0.02), CAMHS externalising disorders with both AMHS neurotic disorders (χ^2^ = 5.02, *P* = 0.03) and EUPD (χ^2^ = 8.07, *P* < 0.001), and CAMHS depression and AMHS externalising disorder (χ^2^ = 6.08, *P* = 0.02).

There were two new cases of schizophrenia in the AMHS sample. The participants had diagnoses of emotional difficulties and externalising disorder while under CAMHS. Of the three new cases of bipolar affective disorder, one participant had a diagnosis of externalising disorder and two had a diagnosis of depression while under CAMHS.

## Discussion

In our sample, the majority of participants experienced a change in diagnosis between adolescence and adulthood. The aim of this study was to explore the patterns of this change. A total of 59% of participants recovered from their CAMHS diagnosis, indicating effective treatment or evolving symptoms. After transitioning to AMHS, 75% of all participants had been diagnosed with a new diagnosis. This highlights that comorbidity and complexity is still developing.

Cwm Taf University Health Board has an agreed transition policy between CAMHS and AMHS. Since 2011, patients are referred from CAMHS when they are 17.5 years old. A period of transition is then expected, with joint clinical reviews, until the patient reaches the age of 18. Prior policies recommended the transfer to adult services at age 16 or when formal education ended. Previous research has indicated that only 49% of CAMHS patients that have reached the age of service transition successfully engage with AMHS.^11^ If these individuals had a history of severe mental illness, prescriptions or previous admissions they were more likely to transition. Our study includes people who have been assessed and diagnosed by both CAMHS and AMHS. In this context, it is therefore difficult to establish if the sample is representative as comparable research has mostly been done on a population level. This may explain why several disorders displayed an inverse relationship although research indicates a comorbid relationship.^8^^,^^10^^,^^12^ In our sample, externalising disorders were more prevalent than depression. This is a possible explanation for the higher proportion of male participants in our sample.^13^ This is important as females are at a higher risk for adolescent mental illness continuing into adulthood^6^ and are more likely to develop comorbid mental disorders than males.^14^

Homotypic continuity into adulthood was statistically present for several disorders, including externalising disorders. This was an expected finding as attention-deficit hyperactivity disorder has an accepted degree of diagnostic stability, continuing into adulthood with population rates ranging from 1.2 to 7.3%.^15^ Neurotic disorders also continued into adulthood. This supports research showing adolescent depression and neurotic disorders displaying a modest continuity into adulthood, in a relapsing and remitting pattern.^4^^,^^16^ This pattern may have influenced why neurotic disorders, but not depression, showed homotypic continuity in this cross-sectional study. Homotypic continuity was also evident for bipolar disorder. Despite this being a small sample, the finding is in keeping with research showing bipolar disorder to be relatively stable diagnostically.^17^ Homotypic continuity was also present for pervasive developmental disorder. This is to be expected as it is considered a lifelong diagnosis.

All CAMHS participants with anorexia nervosa had recovered, and 60% developed EUPD. The DSM defines borderline personality disorder (BPD) instead of EUPD. As EUPD and BPD are comparable, research into both disorders may provide insights into their relationship with eating disorders. Eating disorders have been reported in histories of people with BPD at a rate of 54%, a percentage comparable with our findings.^18^ Difficulty in mood regulation, less distress tolerance and a history of childhood emotional abuse are shared findings for these disorders.^19^^–^^22^ Identifying such shared characteristics may be the key to developing an understanding of which individual's disorders progress and which resolve.

Within the EUPD population in AMHS, six participants (50%) had a history of emotional difficulties in their childhoods. In the ICD-10, diagnostic categories separate children and adults at times. It could therefore be argued that homotypic continuity be considered despite a change in diagnosis. However, we would argue the existence of these age-related restrictions highlights that symptoms are different at different developmental stages. It is important to note that no diagnostic criteria for child- or adult-specific disorders are the same, minus the age restriction. For this reason, CAMHS emotional difficulties progressing to AMHS EUPD was considered a heterotypic transition. Similarly, externalising disorders transitioning to DPD was considered a heterotypic transition. This is despite the frequency of this particular diagnostic transition.^23^ Research has shown that particular traits make the transition to DPD more frequent.^24^ These traits include persistent conduct problems and engaging in more victim-orientated and violent offences. In our sample, 15% of CAMHS externalising disorders progressed to DPD. All of these participants were initially diagnosed with conduct disorder. As antisocial behaviour peaks in adolescence, this could be considered the ideal time to target intervention, especially as research indicates poor outcomes when antisocial behaviour is related to conduct disorder and substance misuse.^25^

Two cases of adolescent depression developed into bipolar disorder. Bipolar disorder is commonly diagnosed within the first 5 years after the first depressive episode.^26^^,^^27^ This time frame highlights diagnostic progression during the transition to adulthood. Prodromal syndromes, preceding schizophrenia, are also often associated with this epoch. These non-specific changes to a person's mental state are often assumed to be normal behaviour or mental disorders, such as depression or anxiety.^28^ It is debatable as to whether these adolescent diagnoses should be seen as displaying heterotypic change or are better characterised as prodromal syndromes. Although prodromal symptoms may be present in the childhoods of people with schizophrenia, diagnosable mental health difficulties may also be present. It is hoped that further research in this area will benefit from the DSM-5 (2013) inclusion of attenuated psychosis syndrome.

This study has highlighted patterns of disorder transition as individuals move from adolescence to adulthood. A developmental perspective of mental illness would postulate that these disorders develop from common vulnerabilities, displaying different symptoms at different developmental stages.^1^^,^^4^ Research has also indicated diagnostic transition peaks in adolescence and early adulthood.^4^^,^^9^^,^^10^ Future research, focusing on adolescence and young adulthood as the formative years for mental health, could facilitate an early identification of individuals at risk and the development of targeted interventions.

Owing to this study's sample size, there is a limit on how generalisable the results are to other mental health teams. This study also contained young people, on average aged 18, and over half had transitioned within 2 years. Follow-up until the mid-twenties may have better captured the diagnostic change during the at-risk period of early adulthood.^6^^,^^29^ It is assumed that the standard of practice would have been similar among the CAMHS professionals as they worked for the same organisation and used the same diagnostic criteria, and the same is assumed for the AMHS professionals. However, bias may have been introduced into this study during the transition from CAMHS and AMHS. CAMHS services were developed using the Common Assessment Framework or the Choice and Partnership Approach, with a focus on formulation. Care and Treatment Plans, with a diagnostic focus, were more common in AMHS. In Wales, both teams are now required to produce a Care and Treatment Plan in keeping with Part 2 of the Mental Health Measure.^30^ This was made law in 2012 and one of the key aims is that it would improve consistency. As this study captures a predominately earlier cohort, it would be interesting to see if legislation has had its desired effect and if bias in diagnosis is now less of a concern. In our opinion, further research would benefit from a larger sample size, longer follow-up and structured interviews by blinded researchers.

## About the authors

**Ann Collins** is a Child and Adolescent Mental Health Services Speciality Registrar at the Tonteg Child and Family Clinic, Cwm Taf University Health Board, Pontypridd, Wales, UK. **Antonio Muñoz-Solomando** is a Consultant Child and Adolescent Mental Health Services Psychiatrist at the Tonteg Child and Family Centre, Cwm Taf University Health Board, Pontypridd, Wales, UK.
